# Prediction of disease-related miRNAs by voting with multiple classifiers

**DOI:** 10.1186/s12859-023-05308-x

**Published:** 2023-04-30

**Authors:** Changlong Gu, Xiaoying Li

**Affiliations:** College of Information Science and Engineering, Hunan University, Changsha, 410082 Hunan China

**Keywords:** miRNA similarity, Disease similarity, Multi-classifiers voting, Cross-validation, XGBoost classification, Random forest classification

## Abstract

There is strong evidence to support that mutations and dysregulation of miRNAs are associated with a variety of diseases, including cancer. However, the experimental methods used to identify disease-related miRNAs are expensive and time-consuming. Effective computational approaches to identify disease-related miRNAs are in high demand and would aid in the detection of lncRNA biomarkers for disease diagnosis, treatment, and prevention. In this study, we develop an ensemble learning framework to reveal the potential associations between miRNAs and diseases (ELMDA). The ELMDA framework does not rely on the known associations when calculating miRNA and disease similarities and uses multi-classifiers voting to predict disease-related miRNAs. As a result, the average AUC of the ELMDA framework was 0.9229 for the HMDD v2.0 database in a fivefold cross-validation. All potential associations in the HMDD V2.0 database were predicted, and 90% of the top 50 results were verified with the updated HMDD V3.2 database. The ELMDA framework was implemented to investigate gastric neoplasms, prostate neoplasms and colon neoplasms, and 100%, 94%, and 90%, respectively, of the top 50 potential miRNAs were validated by the HMDD V3.2 database. Moreover, the ELMDA framework can predict isolated disease-related miRNAs. In conclusion, ELMDA appears to be a reliable method to uncover disease-associated miRNAs.

## Background

Identification of disease-related biomarkers and the interaction of biomolecules is an emerging and challenging task [1–3]. Many effective methods have been proposed by scholars in different fields [4–6], and the recognition of disease-related microRNAs (miRNAs) is one of the important branches. MiRNAs are small single-stranded non-coding RNA molecules (containing approximately 22 nucleotides) that can regulate gene expression at the posttranscriptional level [7]. MiRNAs play an important role in multiple biological processes, including cellular differentiation, proliferation, apoptosis and tissue development [8]. Substantial evidence indicates that miRNA dysregulation is related to a number of human diseases, such as cardiovascular disease, schizophrenia, and cancer [9]. Thus, the identification of disease-related miRNAs will be helpful in the diagnosis, treatment, and prevention of diseases.

Through biological experiments, such as Northern hybridization, microarray analysis, and real-time quantitative PCR, scientists have verified a large number of miRNA-disease associations [10]. By collecting and sorting miRNA-disease associations, Cui et al. constructed a comprehensive database, namely, the Human miRNA-associated Disease Database (HMDD) [11, 12]. The current version is HMDD V3.2; they manually collected 35,547 miRNA-disease association entries, which included 1206 miRNA genes and 893 diseases from 19,280 papers. In addition, in 2010, the team of Andrew E. produced the first release of dbDEMC, which represents a database for collecting differentially expressed miRNAs in human cancers obtained from microarray data [13]. Since then, they have maintained and updated the database, and the latest version is dbDEMC 3.0. This current version contains 3268 differentially expressed miRNAs from 40 cancer types, whereas for humans, a total of 2584 differentially expressed miRNAs were included. Focusing on different studies, there are many miRNA-related databases that provide a strong data source for miRNA research.

The identification of disease-related miRNAs by biological experimental methods has high costs and takes a long time, so effective calculation methods for predicting disease-related miRNAs have attracted extensive attention. In the past few years, significant progress has been made in the development of miRNA disease association prediction models. These models can be roughly divided into two categories: models based on score functions, models based on network algorithms or models based on machine learning.

Most methods that predict miRNA disease associations based on score functions are based on the assumption that functionally similar miRNAs tend to be associated with phenotypically similar diseases [14]. Xuan et al. [15] presented the HDMP prediction model based on the most highly weighted similar neighbors to predict potential miRNA-disease associations. The model combined the information content of disease terms and phenotype similarities among diseases to calculate miRNA functional similarities and used miRNA family information to further improve the prediction accuracy. However, this method will fail when miRNA has no known associated diseases. Chen et al. [16] developed a computational model named WBSMDA to predict disease-related miRNAs by integrating known miRNA-disease associations, miRNA functional similarities, disease semantic similarities and Gaussian interaction profile kernel similarities. This method obtains the final prediction scores by integrating Within-Scores and Between-Scores, which are used for miRNA disease association predictions, achieves a good prediction effect, and can be applied to diseases without any known related miRNAs and miRNAs without any known related diseases.

Some researchers predict disease-related miRNAs based on network algorithms, such as network embedding, network projection, matrix factorization, and random walk. These methods construct similarity networks of miRNAs and diseases from different perspectives and then implement network algorithms to predict the associations among miRNAs and diseases. For example, by integrating known human miRNA–disease associations, miRNA similarities and disease similarities, You et al. [17] proposed a path-based computational model for miRNA–disease association predictions. They constructed a heterogeneous graph with many paths and used the sum of all path scores to calculate the association probabilities of miRNA-disease pairs. Due to the sparsity of the known miRNA disease association matrix, this affects the performance of this model. Chen et al. [18] presented a prediction model of bipartite network projection for miRNA–disease association prediction (BNPMDA). Based on the known miRNA–disease association network, miRNA similarity network and disease similarity network, they constructed bias ratings for miRNAs and diseases and implemented a bipartite network recommendation algorithm to predict disease-related miRNAs. Recently, Chen et al. [19] developed a neoteric Bayesian model to predict potential miRNA-disease associations, named KBMFMDA, which combines kernel-based nonlinear dimensionality reduction, matrix factorization and binary classification. Based on random walk and binary regression, Niu et al. [20] presented a prediction model using RWBRMDA, which extracted the features of miRNAs by a random walk with restart, and applied binary logistic regression to calculate the probability scores of miRNA-disease pairs. The limitation of RWBRMDA is that it cannot predict new diseases that have no known related miRNAs. The analysis of biological molecular data related to diseases is highly complex, and examining the data from various perspectives can aid in comprehending the pathogenesis of diseases. Consequently, multi-network integrated learning models have emerged as a promising approach and have yielded favorable outcomes [21–24]. For example, Ma et al. [24] proposed a computational model, DeepMNE, which employs deep multi-network embedding to integrate multi-omics data and identify potential lncRNA-disease associations. Both cross-validation and case studies have demonstrated the excellent predictive performance of DeepMNE.

In recent years, the method of predicting disease-related miRNAs based on machine learning has appeared in a blowout. Machine-based learning methods predict disease-related miRNAs through a trained model. The training model needs the characteristics and labels of positive and negative samples. Therefore, the problems of feature selection and negative samples need to be solved. Chen et al. [25] developed a ranking-based k-nearest neighbor calculation method of RKNNMDA to predict disease-related miRNAs. By combining miRNA similarities, disease similarities, Gaussian kernel similarities and known miRNA disease associations, the K-nearest neighbors (KNN) algorithm is used to search the nearest K neighbors of miRNA and disease. Then, these k-nearest neighbors are reranked according to the SVM ranking model. Finally, the ranking results are weighted to obtain the final ranking of all potential miRNA-disease associations. The disadvantage of RKNNMDA is that it may be biased toward miRNAs with more known related diseases. Peng et al. [26] proposed a learning-based framework, MDA-CNN, for miRNA-disease association identification. The model extracts features by using an autoencoder based on three networks with an additional target gene layer, inputs the features into a CNN and identifies disease-connected miRNAs. Considering the difficulty in obtaining negative samples, Chen et al. [27] proposed a semi-supervised model to predict miRNA disease associations. This model applied the regularized least squares (RLS) method to construct two optimal classifiers based on miRNA functional similarities and disease semantic similarities and can be applied to new diseases that have no associated miRNAs. However, this method must manually adjust the parameters to balance the contributions of the two classifiers. Chen et al. [28] implemented ensemble learning models, named EDTMDA, to distinguish potential associations from unknown miRNA-disease associations. EDTMDA fuses multiple basic classifiers to infer novel miRNA-disease associations, which achieves good prediction accuracy. Due to the rapidity and effectiveness of unstructured data processing, deep learning methods are widely used in miRNA-disease association predictions. For example, CNNMDA [29] utilized dual convolutional neural networks (CNNs) to learn the original and global representations of miRNA–disease pairs. However, machine learning-based algorithms face difficulties in retrieving negative samples, which may decrease their prediction performance. Recently, hypergraph learning has been used to identify disease-related biomarkers [30–32]. Based on attention aware multi-view similarity networks and hypergraph learning, Ning et al. [32] developed a model called AMHMDA for identifying disease-related miRNAs. The experimental results have shown that AMHMDA has good performance, and the case study further confirms the predictive ability of the model.

In this work, we propose an ensemble learning framework for miRNA disease association prediction, named ELMDA. The ELMDA framework integrates miRNA and disease similarities along with known miRNA-disease associations to reveal potential miRNA-disease associations. The main contributions of the paper are summarized as follows.

1. The target data verified by experiments are used to construct similarity networks, which can avoid false-positives of target data.
2. Both disease and miRNA similarity network construction do not consider the known association data, and cross validation can avoid overestimating the prediction performance of the model.
3. The ELMDA framework extracts features from similarity data to reduce the data scale, adds structural feature data to obtain more complete data features, and selects appropriate negative samples through sample selection so that the model has good prediction performance.
4. The ELMDA framework uses multiple classifiers to vote for the final prediction, and the model has good generalization ability.
5. The ELMDA framework can be applied to predict isolated diseases (diseases without any known related miRNAs).

## Results

### Performance of ELMDA based on fivefold cross-validation

In this section, to validate the ability of ELMDA to predict potential miRNA-disease associations, we adopt fivefold cross-validation in our experiment. The training dataset is randomly and evenly divided into five subsets; then, one subset is used for testing, and the other four subsets are selected for training. This process is repeated until all subsets have been used as the test set. We assessed the performance of the methods using the following evaluation criteria: precision [Eq. (1)], recall [Eq. (2)] and F1-score [Eq. (3)]. The formulas are as follows:

1 $$precision= \frac{TP}{TP+FP}$$

2 $$recall= \frac{TP}{TP+FN}$$

3 $$F1-score= \frac{2*precision*recall}{precision+recall}$$

where TP and TN represent the number of correctly identified positive and negative samples respectively, FP and FN represent the number of false positive and false negative samples. In addition, we draw receiver operating characteristic curve (ROC) and use the area under the curve (AUC) to evaluate these methods. The ROC curve plots true-positive rate (TPR) versus false-positive rate (FPR) at different thresholds. However, due to the small number of positive samples (experimentally verified miRNA-disease associations), using only the AUC to evaluate the performance was too arbitrary; thus, we also used the precision-recall (PR) curve and area under the PR curve (AUPR) to complement the performance evaluation. In general, if the ROC and PR curves show similar variations and the AUC and AUPR values are close to 1, the prediction performance is better.

**Fig. 1:**
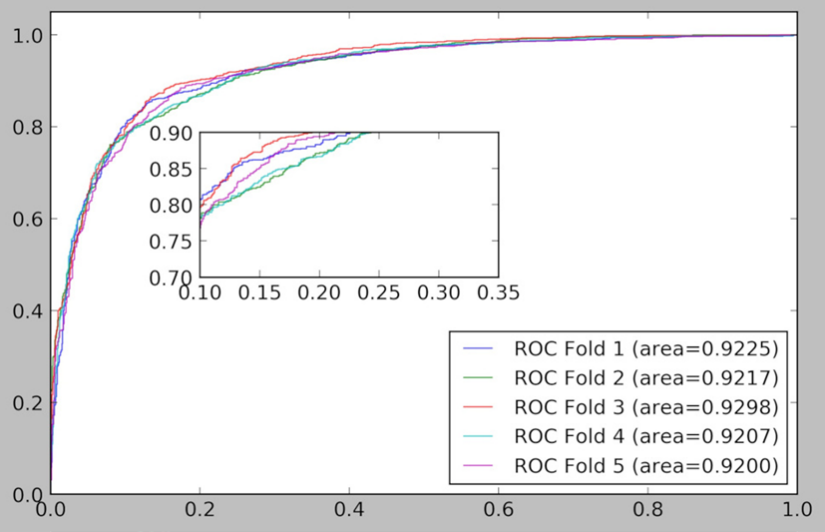
ROC curve and the local enlarged figure of the ELMDA framework

**Fig. 2:**
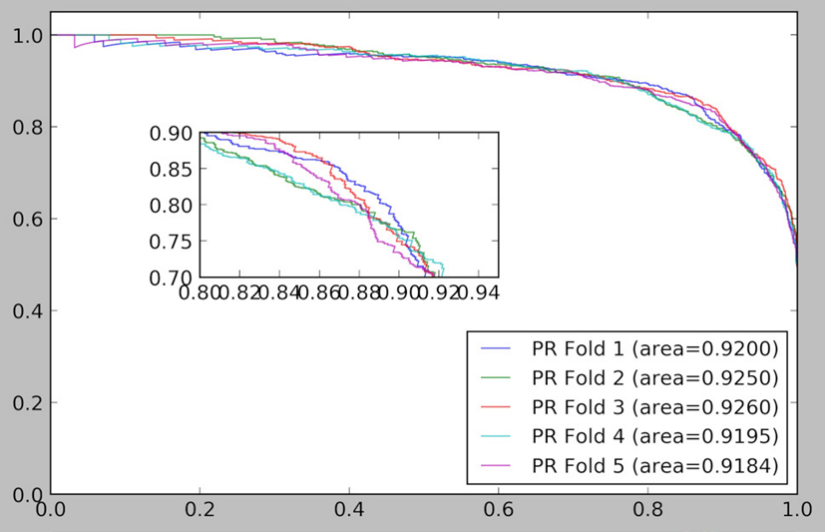
PR curve and the local enlarged figure of the ELMDA framework

The fivefold cross validation results of the ELMDA framework are shown in Table 1. The ELMDA framework clearly exhibits a commendable predictive performance with an average AUC value of 0.9229. The maximum AUC value is 0.9299, and the minimum AUC value is 0.9207. The ROC curve and PR curve and the local enlarged figure of the ELMDA framework are shown in Figs. 1 and 2, respectively. Based on these results, the ELMDA framework shows good prediction performance.

**Table 1:** Fivefold cross validation results of the ELMDA framework

Fold	Precision	Recall	F1-score	AUC	AUPR
1	0.8709	0.8469	0.8587	0.9225	0.9200
2	0.8359	0.8412	0.8386	0.9216	0.9250
3	0.8544	0.8704	0.8623	0.9298	0.9261
4	0.8326	0.8510	0.8417	0.9207	0.9195
5	0.8463	0.8579	0.8521	0.9201	0.9184
Average	0.8480 ± 0.0138	0.8535 ± 0.0101	0.8507 ± 0.0093	0.9229 ± 0.0035	0.9218 ± 0.0031

The ELMDA framework combines four classification algorithms: SVM, GBDT, RF and XGBoost. Next, we compare the performance of a single classifier and the ELMDA framework. The results are shown in Table 2. For all prediction performance evaluation indicators, including Precision, Recall, F1-score, AUC and AUPR, the ELMDA framework is superior to the prediction performance of a single classifier. Therefore, the construction of the ELMDA framework is reasonable and can improve the overall prediction ability.

**Table 2:** Comparison of the prediction performance of the ELMDA framework and a single classifier

Fold	Precision	Recall	F1-score	AUC	AUPR
SVM	0.8369 ± 0.0085	0.8371 ± 0.0143	0.8370 ± 0.0075	0.9091 ± 0.0031	0.9057 ± 0.0036
GBDT	0.8369 ± 0.0107	0.8490 ± 0.0057	0.8429 ± 0.0054	0.9172 ± 0.0034	0.9138 ± 0.0039
RF	0.8424 ± 0.0108	0.8354 ± 0.0131	0.8388 ± 0.0091	0.9141 ± 0.0049	0.9123 ± 0.0047
XGboost	0.8471 ± 0.0090	0.8486 ± 0.0099	0.8478 ± 0.0076	0.9191 ± 0.0039	0.9165 ± 0.0045
ELMDA	0.8485 ± 0.0139	0.8536 ± 0.0101	0.8510 ± 0.0094	0.9229 ± 0.0035	0.9217 ± 0.0031

### Comparison with other methods

**Fig. 3:**
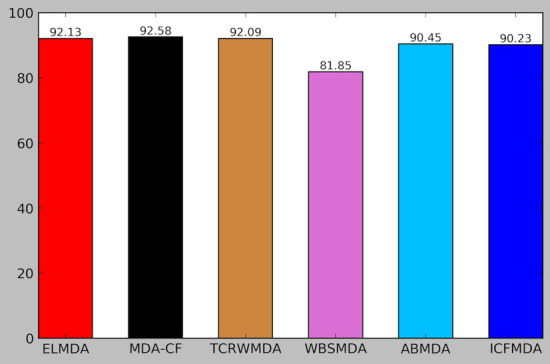
Comparison of the performance of the ELMDA framework with other new computing methods

We compared the performance of the ELMDA framework with other novel computational methods, including MDA-CF[30], TCRWMDA [31], WBSMDA [32], ABMDA [33] and ICFMDA[34]. Like ours, these methods are also developed based on HMDD V2.0, and are all based on five-fold cross validation and evaluated using AUC values. Each group is selected as the test set in turn, and the other 4 groups of data are used for training. The predicted scores of the test samples were obtained, and the scores of all miRNA-disease pairs were ranked. Then, we calculated TPRSs and FPRs at different thresholds and obtained AUCs. The whole procedure was repeated 20 times to obtain the average results. The results are shown in Fig. 3. The predicted AUCs of the six computational models were 92.13, 92.58, 92.09, 81.85, 90.45, and 90.23, respectively. The AUC of the ELMDA framework is slightly lower than that of MDA-CF and better than those of the other four methods. Without using known association data, the ELMDA framework achieves satisfactory performance, while other algorithms use known association data. The results further confirmed the efficiency of the ELMDA framework for miRNA-disease association predictions.

## Case study

To investigate the ability of the ELMDA framework to infer unknown miRNA-disease associations, we implemented case studies from two different perspectives. We first evaluated the overall model performance, trained the model with 5430 known associations present in the HMDD V2.0 dataset as positive samples and 5418 selected negative samples, and then predicted unknown associations (candidate associations) in the HMDD V2.0 dataset, sorted the prediction results, selected the top 50 candidate associations with the highest rankings, and verified them with HMDD V3.2 (the latest version). The predicted results are presented in Table 3. Among the top 50 predicted associations, only five have not been confirmed by the HMDD V3.2 database, and the top 10 were all confirmed. Among the prediction results not verified by HMDD V3.2, the 29th and 50th results found new evidence support through literature search. Based on genome analysis, Anna Barbato et al. [33] found that melanoma tissues with high hsa-mir-181a and hsa-mir-181b expression presented favorable outcomes in terms of progression free survival, suggesting that has-mir-181 is a clinically relevant candidate for biomarker-based therapy selection. Wu et al. [34] suggest that miR-93-5p modulates tumorigenesis and gemcitabine resistance in pancreatic cancer cells via targeting the PTEN/PI3K/Akt signaling pathway.

**Table 3:** Predictions of all potential associations in the HMDD V2.0 database and 90% of the top 50 results are verified by the updated HMDD V3.2 database

rank	miRNA	Disease	Validated
1	hsa-mir-16	Lung neoplasms	True
2	hsa-mir-155	Glioblastoma	True
3	hsa-mir-21	Stomach neoplasms	True
4	hsa-mir-29a	Pancreatic neoplasms	True
5	hsa-mir-17	Carcinoma, renal cell	True
6	hsa-mir-155	Prostatic neoplasms	True
7	hsa-mir-9	Carcinoma, hepatocellular	True
8	hsa-mir-150	Breast neoplasms	True
9	hsa-mir-20a	Carcinoma, renal cell	True
10	hsa-mir-142	Breast neoplasms	True
11	hsa-mir-106b	Lung neoplasms	True
12	hsa-mir-155	Stomach neoplasms	True
13	hsa-mir-21	Retinoblastoma	True
14	hsa-mir-145	Stomach neoplasms	True
15	hsa-mir-34a	Heart failure	True
16	hsa-mir-98	Breast neoplasms	True
17	hsa-mir-155	Autistic disorder	False
18	hsa-mir-126	Stomach neoplasms	True
19	hsa-mir-155	Glioma	True
20	hsa-mir-125b	Heart failure	True
21	hsa-mir-21	Nasopharyngeal Neoplasms	True
22	hsa-mir-15b	Lung neoplasms	True
23	hsa-mir-17	Stomach neoplasms	True
24	hsa-mir-34b	Carcinoma, hepatocellular	True
25	hsa-mir-205	Colorectal neoplasms	True
26	hsa-mir-21	Hepatitis b	True
27	hsa-mir-15a	Lung neoplasms	True
28	hsa-mir-130a	Breast neoplasms	True
29	hsa-mir-181b	Melanoma	False
30	hsa-mir-29b	Colorectal neoplasms	True
31	hsa-mir-221	Heart failure	True
32	hsa-mir-195	Lung neoplasms	True
33	hsa-mir-20a	Stomach neoplasms	True
34	hsa-mir-98	Melanoma	True
35	hsa-mir-101	Melanoma	True
36	hsa-mir-9	Heart failure	False
37	hsa-mir-214	Colorectal neoplasms	True
38	hsa-mir-29a	Stomach neoplasms	True
39	hsa-mir-21	Schizophrenia	False
40	hsa-mir-21	Carcinoma, basal cell	True
41	hsa-mir-122	Lung neoplasms	True
42	hsa-mir-223	Melanoma	True
43	hsa-mir-17	Carcinoma	True
44	hsa-mir-378a	Breast neoplasms	True
45	hsa-mir-29c	Colorectal neoplasms	True
46	hsa-mir-143	Carcinoma, Hepatocellular	True
47	hsa-mir-222	Heart failure	True
48	hsa-mir-1	Neoplasms	True
49	hsa-mir-29a	Glioblastoma	True
50	hsa-mir-93	Pancreatic neoplasms	False

Furthermore, the same strategy was adopted for specific diseases, and three case studies were carried out on gastric neoplasms, prostate neoplasms and colon neoplasms. As shown in Table 4, 50, 47 and 45 of the top 50 miRNAs, respectively, predicted by the ELMDA framework were validated by HMDD V3.2.

**Table 4:** The ELMDA framework was implemented to investigate gastric neoplasms, prostate neoplasms and colon neoplasms, and 100%, 94%, and 90%, respectively, of the top 50 potential miRNAs were validated by HMDD V3.2

Rank	Gastric neoplasms		PROSTATE neoplasms		COLON neoplasms	
	miRNA	Valided	miRNA	Valided	miRNA	Valided
1	hsa-mir-21	True	hsa-mir-21	True	hsa-mir-20a	True
2	hsa-mir-146a	True	hsa-mir-155	True	hsa-mir-93	True
3	hsa-mir-155	True	hsa-mir-34a	True	hsa-mir-21	True
4	hsa-mir-29a	True	hsa-mir-29a	True	hsa-mir-29a	True
5	hsa-mir-145	True	hsa-mir-222	True	hsa-mir-155	True
6	hsa-mir-17	True	hsa-mir-18a	True	hsa-mir-146a	True
7	hsa-mir-126	True	hsa-mir-146a	True	hsa-mir-122	True
8	hsa-mir-20a	True	hsa-mir-29b	True	hsa-mir-125b	True
9	hsa-mir-29b	True	hsa-mir-221	True	hsa-mir-221	True
10	hsa-mir-125b	True	hsa-mir-17	True	hsa-mir-106b	True
11	hsa-mir-200b	True	hsa-mir-122	True	hsa-mir-29b	True
12	hsa-mir-222	True	hsa-mir-20a	True	hsa-mir-182	True
13	hsa-mir-18a	True	hsa-mir-34c	True	hsa-mir-222	True
14	hsa-mir-221	True	hsa-mir-34b	True	hsa-mir-34a	True
15	hsa-mir-200c	True	hsa-mir-199a	True	hsa-mir-29c	True
16	hsa-mir-29c	True	hsa-mir-210	True	hsa-mir-20b	True
17	hsa-mir-1	True	hsa-mir-133b	True	hsa-mir-199a	True
18	hsa-mir-146b	True	hsa-mir-223	True	hsa-mir-205	True
19	hsa-mir-93	True	hsa-mir-93	True	hsa-mir-214	True
20	hsa-mir-107	True	hsa-mir-126	True	hsa-mir-34b	True
21	hsa-mir-9	True	hsa-mir-124	True	hsa-mir-34c	True
22	hsa-mir-34a	True	hsa-mir-184	False	hsa-mir-200b	True
23	hsa-mir-182	True	hsa-mir-214	True	hsa-mir-133a	True
24	hsa-mir-26a	True	hsa-mir-182	True	hsa-mir-200c	True
25	hsa-mir-23b	True	hsa-mir-27a	True	hsa-mir-18a	True
26	hsa-mir-34b	True	hsa-mir-29c	True	hsa-mir-31	True
27	hsa-let-7a	True	hsa-mir-342	True	hsa-mir-146b	False
28	hsa-mir-133b	True	hsa-mir-99b	True	hsa-mir-183	True
29	hsa-mir-27a	True	hsa-mir-23a	True	hsa-mir-486	True
30	hsa-mir-34c	True	hsa-mir-486	True	hsa-mir-107	True
31	hsa-let-7c	True	hsa-mir-133a	True	hsa-mir-133b	True
32	hsa-let-7b	True	hsa-mir-31	True	hsa-mir-23a	True
33	hsa-mir-106b	True	hsa-mir-200b	True	hsa-mir-223	True
34	hsa-mir-133a	True	hsa-mir-92a	True	hsa-mir-140	True
35	hsa-mir-183	True	hsa-mir-192	True	hsa-mir-143	True
36	hsa-mir-214	True	hsa-mir-16	True	hsa-mir-519d	False
37	hsa-mir-342	True	hsa-mir-224	True	hsa-let-7b	True
38	hsa-mir-200a	True	hsa-mir-7	True	hsa-mir-9	False
39	hsa-mir-196a	True	hsa-mir-146b	True	hsa-mir-1	True
40	hsa-mir-31	True	hsa-mir-200c	True	hsa-mir-124	False
41	hsa-mir-122	True	hsa-mir-151a	True	hsa-mir-125a	True
42	hsa-mir-181a	True	hsa-mir-137	False	hsa-mir-210	True
43	hsa-mir-16	True	hsa-mir-9	True	hsa-mir-429	True
44	hsa-let-7g	True	hsa-mir-429	True	hsa-mir-101	True
45	hsa-mir-142	True	hsa-mir-1	True	hsa-mir-96	True
46	hsa-mir-223	True	hsa-mir-205	True	hsa-mir-7	False
47	hsa-mir-206	True	hsa-mir-423	False	hsa-mir-10b	True
48	hsa-mir-141	True	hsa-mir-96	True	hsa-mir-130a	True
49	hsa-mir-101	True	hsa-mir-200a	True	hsa-mir-218	True
50	hsa-mir-486	True	hsa-mir-106b	True	hsa-let-7a	True

An isolated disease refers to a disease without any known associated miRNA. To further evaluate the predicted performance of the ELMDA framework for predicting isolated disease-related miRNAs, the predicted scores of potential associations were calculated by removing all known associations related to predicted diseases. Isolated disease-related miRNA predictions were implemented for gastric neoplasms, prostate neoplasms and colon neoplasms. As shown in Table 5, 48, 42 and 43, respectively, of the top 50 miRNAs predicted by the ELMDA framework were validated by HMDD V3.2.

**Table 5:** Predictions of isolated disease-related miRNAs for gastric neoplasms, prostate neoplasms and colon neoplasms; 96%, 84%, and 86%, respectively, of the top 50 potential miRNAs were validated by HMDD V3.2

rank	Gastric neoplasms		Prostate neoplasms		Colon neoplasms	
	miRNA	Valided	miRNA	Valided	miRNA	Valided
1	hsa-mir-21	True	hsa-mir-17	True	hsa-mir-21	True
2	hsa-mir-146a	True	hsa-mir-21	True	hsa-mir-29a	True
3	hsa-mir-155	True	hsa-mir-20a	True	hsa-mir-221	True
4	hsa-mir-17	True	hsa-mir-122	True	hsa-mir-155	True
5	hsa-mir-145	True	hsa-mir-29a	True	hsa-mir-122	True
6	hsa-mir-20a	True	hsa-mir-146a	True	hsa-mir-222	True
7	hsa-mir-125b	True	hsa-mir-93	True	hsa-mir-146a	True
8	hsa-mir-93	True	hsa-mir-133b	True	hsa-mir-34a	True
9	hsa-mir-29b	true	hsa-mir-34a	True	hsa-mir-29c	True
10	hsa-mir-222	True	hsa-mir-199a	True	hsa-mir-133b	True
11	hsa-mir-29a	True	hsa-mir-29c	True	hsa-mir-18a	True
12	hsa-mir-1	True	hsa-mir-155	True	hsa-mir-146b	False False
13	hsa-mir-221	True	hsa-mir-210	True	hsa-mir-29b	True
14	hsa-mir-133b	True	hsa-mir-200a	True	hsa-mir-223	True
15	hsa-mir-18a	True	hsa-mir-34c	True	hsa-mir-125b	True
16	hsa-mir-34a	True	hsa-mir-184	False	hsa-mir-486	True
17	hsa-mir-126	True	hsa-mir-29b	True	hsa-mir-34c	True
18	hsa-mir-27a	True	hsa-mir-126	True	hsa-mir-151a	False
19	hsa-mir-106b	True	hsa-mir-151a	True	hsa-mir-23a	True
20	hsa-let-7b	True	hsa-mir-18a	True	hsa-mir-34b	True
21	hsa-let-7a	True	hsa-mir-192	True	hsa-mir-133a	True
22	hsa-mir-29c	True	hsa-mir-222	True	hsa-mir-107	True
23	hsa-mir-16	True	hsa-mir-31	True	hsa-mir-210	True
24	hsa-mir-146b	True	hsa-mir-106b	True	hsa-mir-200c	True
25	hsa-mir-9	True	hsa-mir-200b	True	hsa-mir-214	True
26	hsa-mir-196a	True	hsa-mir-133a	True	hsa-mir-99a	True
27	hsa-mir-26a	True	hsa-mir-215	False	hsa-mir-200b	True
28	hsa-let-7c	True	hsa-mir-151b	True	hsa-mir-31	True
29	hsa-mir-206	True	hsa-mir-199b	False	hsa-mir-182	True
30	hsa-mir-124	True	hsa-mir-20b	True	hsa-mir-199a	True
31	hsa-mir-183	True	hsa-mir-429	True	hsa-mir-23b	True
32	hsa-mir-373	True	hsa-mir-146b	True	hsa-mir-9	False
33	hsa-mir-27b	True	hsa-mir-141	True	hsa-mir-183	True
34	hsa-mir-142	True	hsa-mir-34b	True	hsa-mir-96	True
35	hsa-mir-122	True	hsa-mir-1	True	hsa-mir-205	True
36	hsa-mir-98	False	hsa-mir-28	False	hsa-mir-137	True
37	hsa-mir-214	True	hsa-mir-371a	False	hsa-mir-342	False
38	hsa-mir-15b	True	hsa-mir-137	False	hsa-mir-429	True
39	hsa-mir-34b	True	hsa-mir-148a	True	hsa-mir-103a	True
40	hsa-mir-34c	True	hsa-mir-200c	True	hsa-mir-143	True
41	hsa-mir-107	True	hsa-mir-451a	False	hsa-mir-27a	True
42	hsa-mir-133a	True	hsa-mir-486	True	hsa-mir-150	True
43	hsa-let-7e	False	hsa-mir-449a	True	hsa-mir-708	False
44	hsa-mir-181a	True	hsa-mir-106a	True	hsa-mir-326	True
45	hsa-let-7g	True	hsa-mir-203	True	hsa-mir-124	False
46	hsa-mir-200b	True	hsa-mir-182	True	hsa-mir-10b	True
47	hsa-mir-20b	True	hsa-mir-185	True	hsa-mir-99b	True
48	hsa-mir-205	True	hsa-mir-224	True	hsa-mir-130a	True
49	hsa-mir-342	True	hsa-mir-99b	True	hsa-mir-138	True
50	hsa-mir-101	True	hsa-mir-326	False	hsa-mir-28	False

According to the above description, the ELMDA framework exhibits good performance for predicting potential miRNA-disease associations and isolated disease-related miRNAs.

## Discussion

The accumulating evidence has indicated that miRNAs play important roles in disease development. The identification of disease-related miRNAs will be beneficial to gain a deeper understanding of disease mechanisms at the molecular level. As valuable complements to experimental studies, computational models used to identify associations between miRNAs and diseases are in high demand.

In this work, the miRNA-mRNA interactions verified by experiments are used to construct the miRNA similarity network, and the disease similarity network is constructed by using the similarities of disease functions and disease targets. The training dataset is reconstructed through feature extraction and sample selection, and the model is trained by multiclassifier voting. The model has shown good performance in both the fivefold cross validation and case studies and can predict isolated disease-related miRNAs.

Despite the favorable results obtained using the ELMDA framework, this study has some limitations. First, when we calculated the similarities among miRNAs and diseases, we used target data that were verified by experiments. However, target data that are verified by experiments are very sparse, resulting in no common target genes between many miRNAs and diseases, and the similarities of many miRNA pairs and disease pairs are 0. With the deepening of relevant research, considering that miRNA target genes and disease target genes are increasingly recognized, this problem will improve. Second, the ELMDA framework uses a form of multiclassifier voting to obtain the final prediction scores. We choose the top four classifiers with the highest scores to build the model through experimental methods. There is no theoretical basis for the selection of a single classifier. In the future, we will further study the selection method for classifiers and assign different weights to each classifier to improve the model.

## Conclusion

We propose a model framework, named ELMDA, to predict the unknown miRNA-disease associations. Without considering the known association, the potential association can be predicted by multi-classifiers voting by integrating miRNA and disease similarity network. The performance of the model framework was evaluated through five-fold cross validation, and the predictive ability of the model was verified through case studies. The model can predict miRNAs related to isolated diseases. In conclusion, ELMDA appears to be a reliable method to uncover disease-associated miRNAs.

## Materials and methods

### Human miRNA–disease associations

The experimentally verified human miRNA–disease associations were downloaded from HMDD V2.0 database [11]. The database provides 5430 distinct high-quality experimentally verified miRNA–disease associations, which involve 495 miRNAs and 383 diseases. We use this dataset as the benchmark dataset and variables $\mathrm{m}$ and $\mathrm{d}$ to represent the number of miRNAs and diseases, respectively. The adjacency matrix of miRNA–disease associations is denoted by matrix $A$, whereas the entity $A(i,j)$ in row $i$ and column $j$ is 1 if miRNA $i$ is associated with disease $j$ and 0 otherwise. Matrix $A$ is a very sparse matrix with a known association density of 0.00286. The research task in this work is to discover the potential miRNA-disease associations (0 in matrix $A$).

### Disease similarity and miRNA similarity

Many miRNA disease association prediction models construct miRNA and disease similarity networks, which combine known associations to improve model performance. However, the direct use of these similarity data in the cross validation of the model will overestimate the model performance. If the known association data in the training set are removed and the similarity is recalculated during each cross validation, this will involve great time costs. Considering this factor, we constructed miRNA and disease similarity networks without using known association information.

The disease similarity network consists of two parts: semantic similarity and functional similarity. We use the method proposed by Wang et al. [35] to calculate the disease semantic similarity and use the matrix, SD1, to represent it. SD1(A,B) represents the semantic similarity between disease A and disease B.

Based on the assumption that diseases with similar functions tend to be associated with similar target genes (mRNAs), we measured the functional similarity of the two diseases by considering their related target genes. DisGeNET is a discovery platform containing one of the largest publicly available collections of genes and variants associated with human diseases [36]. Disease-mRNA interactions were obtained from the latest version, DisGeNET V7.0; let ${T}_{d}^{A}=\left\{{T}_{d}^{A}(1),{T}_{d}^{A}(2),\dots ,{T}_{d}^{A}(na)\right\}$ and ${T}_{d}^{B}=\left\{{T}_{d}^{B}(1),{T}_{d}^{B}(2),\dots ,{T}_{d}^{B}(nb)\right\}$ denote the target gene sets of diseases $A$ and $B$, where variables $na$ and $nb$ are the number of target genes of diseases $A$ and $B$, respectively. The information entropy of ${T}_{d}^{A}$ is defined in Eq. (4):

4 $$\left\{\begin{array}{c}H\left({T}_{d}^{A}\right)=-\sum_{i=1}^{na}p\left({T}_{d}^{A}\left(i\right)\right){log}_{2}\left(p\left({T}_{d}^{A}\left(i\right)\right)\right)\\ p\left({T}_{d}^{A}\left(i\right)\right)=\frac{n\left({T}_{d}^{A}\left(i\right)\right)}{N}\end{array}\right.$$

where $N$ is the number of disease-mRNA interactions, $n({T}_{d}^{A}\left(i\right))$ is the number of the $ith$ target gene of disease $A$ in the disease-mRNA set, $p\left({T}_{d}^{A}(i)\right)$ is the frequency of the $ith$ target gene of disease $A$ in the disease-mRNA set, and $H\left({T}_{d}^{A}\right)$ is the information entropy of ${T}_{d}^{A}$.

The normalized mutual information (NMI) of ${T}_{d}^{A}$ and ${T}_{d}^{B}$ is used to measure the functional similarity of diseases $A$ and $B$, as shown in Eq. (5):

5 $$SD2\left(A,B\right)=\frac{2*H\left({T}_{d}^{A}\cap {T}_{d}^{B}\right)}{H\left({T}_{d}^{A}\right)+H\left({T}_{d}^{B}\right)}$$

where $H\left({T}_{d}^{A}\right)$, $H\left({T}_{d}^{B}\right)$ and $H\left({T}_{d}^{A}\cap {T}_{d}^{B}\right)$ represent the information entropy of ${T}_{d}^{A}$, ${T}_{d}^{B}$ and the intersection set of ${T}_{d}^{A}$ and ${T}_{d}^{B}$, respectively. The functional similarity between two diseases is measured by Eq. (5) according to their common target genes and the information entropy of their respective target gene sets and is standardized based on NMI. Matrix $SD2$ is the functional similarity matrix, and $SD2(i,j)$ in row $i$ and column $j$ represents the similarity between diseases $i$ and $j$.

The disease similarity is obtained by integrating the semantic similarity and functional similarity in the Eq. (6):

6 $$SD\left(i,j\right)=\alpha *SD1\left(i,j\right)+\left(1-\alpha \right)*SD2(i,j)$$

where $\alpha$ and $\left(1-\alpha \right)$ are the contribution parameters of the semantic similarity and functional similarity, respectively. In our experiment, it is considered that the contributions are the same, and $\alpha$ is taken as 0.5.

MiRNAs are important regulatory RNAs that mainly function in repressing gene expression at the posttranscriptional level by binding to the 3’-UTR of target mRNAs through base pairing [37]. Based on the assumption that miRNAs with similar functions tend to be associated with similar target genes, we downloaded miRNA‒mRNA interaction data from the miRTarBase database [38, 39], and let ${T}_{m}^{A}=\left\{{T}_{m}^{A}(1),{T}_{m}^{A}(2),\dots ,{T}_{m}^{A}(ma)\right\}$ and ${T}_{m}^{B}=\left\{{T}_{m}^{B}(1),{T}_{m}^{B}(2),\dots ,{T}_{m}^{B}(mb)\right\}$ denote the target gene sets of miRNAs, $A$ and $B$, where variables $ma$ and $mb$ are the number of target genes of miRNA $A$ and $B$, respectively. The MiRNA functional similarities were calculated using the same calculation method as for the disease functional similarities, and the miRNA similarities were represented by matrix $SM$, where $SM(i,j)$ in row $i$ and column $j$ represents the similarity between miRNAs $i$ and $j$.

## ELMDA model

**Fig. 4:**
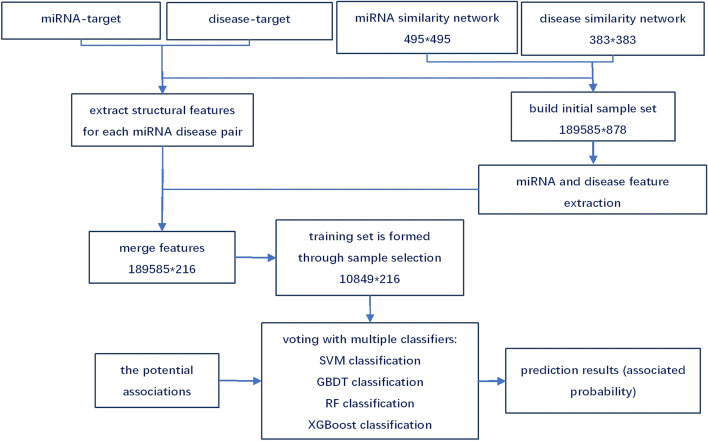
The flowchart of ELMDA model

In this section, we will detail the ELMDA model construction process and show the overall process in Fig. 4.

### Step 1: Structural feature extraction

To more comprehensively describe the sample characteristics and improve the model performance, we extracted the structural features of miRNA and disease and added them to the sample. We extracted 17 structural features for each miRNA disease pair, expressed as $f=\{f\left(1\right),f\left(2\right),\dots ,f(17\}$. $f\left(17\right)$ is the number of target genes associated with miRNA $i$ and disease $j$, which are obtained from the miRTarBase and DisGeNET databases, respectively. The remaining 16 features are composed of two parts. The first 8 features are structural features related to miRNA $i$, and the remaining 8 features are related to disease $j$. The first two structural features of miRNA $i$ are the number and density of miRNA-associated genes. The third and fourth features are the average and variance of the miRNA similarity vector, $SM\left(i,:\right)$, respectively. The remaining four features are the 200 miRNAs most similar to miRNA $i$ and the average similarity calculated for each group of 50 miRNAs. In the same way, the structural characteristics of disease $j$ also include the number and density of disease-associated genes, the mean and variance of disease similarity, and the mean of the most similar disease similarity.

### Step 2: Coding the sample set

Each sample is formulated as $S\left(k\right)=\{SM\left(i,:\right),SD(j,:)\}$, where $SM\left(i,:\right)$ is row $i$ of miRNA similarity matrix $SM$ and $SD(j,:)$ is row $j$ of disease similarity matrix $SD$. The corresponding sample label, $L\left(k\right)$, is 1 if miRNA $i$ is associated with disease $j$; otherwise, the label is 0. The dataset contains 495 miRNAs and 383 diseases, so we obtained a 189,585 × 878 initial sample set $S$. There are a large number of sample features. To improve the efficiency of model training, we reduced the dimensions of the miRNA features and disease features. We used principal component analysis (PCA) dimensionality reduction technology to retain 80% of the data information and finally obtained 80 miRNA features and 119 disease features. Finally, 17 structural features of miRNA and disease were added to each sample (miRNA disease pair) to form the final sample set, which included 189,585 samples and 216 features.

### Step 3: Model architecture

In this work, we used ensembles of machine learning algorithms, such as support vector machine (SVM), gradient boosting decision tree (GBDT), random forest (RF) and eXtreme gradient boosting (XGBoost) classifiers. These algorithms are combined with soft voting classifiers to improve the accuracy and are briefly discussed as follows.

*SVM classification* SVM is a supervised learning algorithm used for classification and regression [40]. An SVM aims to fit an optimal separating hyperplane (OSH) between classes by focusing on the training samples that lie at the edges of the class distributions, the support vectors. A hyperplane is defined as $\omega \bullet x+b=0$, where $x$ is a point lying on the hyperplane, $\omega$ is normal to the hyperplane, and $b$ is the bias. For the linearly separable case, a separating hyperplane can be defined for the two classes as $\omega \bullet {x}_{i}+b\ge +1$ (for ${y}_{i}$= + 1) and $\omega \bullet {x}_{i}+b\le -1$ (for ${y}_{i}$= − 1), where ${y}_{i}$ is the sample category, + 1 is the positive sample and − 1 is the negative sample.

GBDT classification: The gradient advancing decision tree (GBDT) is a machine learning technique used for regression and classification tasks. GBDT provides a prediction model in the form of an integration of weak prediction models (usually decision trees) [41]. When the decision tree is a weak learner, the algorithm generated is called a gradient-boosted tree. In recent years, GBDT has achieved great success in the fields of machine learning and data mining. The gradient-boosted trees model is constructed in the same staged manner as other boosting methods, but it extends other methods by allowing optimization of any differentiable loss function.

*RF classification* Random forest (RF) [42] refers to the establishment of a forest by random sampling. Random refers to random sampling to establish a model; forest means that it consists of many independent decision trees. The basic principle of random forest is as follows: N training datasets are randomly sampled from the original data in the way of putting back; k features are randomly selected from each training dataset (k is less than the total number of features in the original data); M decision trees are established repeatedly according to these K features; each decision tree is applied to predict the results, and the results of all predictions are saved; the classification model is voted on, the number of votes for each prediction result is calculated, and the model with the highest number of votes is selected as the final decision. This method can reduce the risk of overfitting by averaging the decision trees.

*XGBoost classification* XGBoost is a supervised learning algorithm. It implements a process called boosting to generate an accelerated model, which was initially developed by Tianqi Chen [43] and has been further adopted by many developers. Supervised learning is often used to solve classification and regression problems. XGBoost was mainly designed for speed and performance using gradient-boosted decision trees. Boosting is an integrated learning technology that builds multiple models in sequence, and each new model attempts to correct the defects in the previous model. In tree promotion, each new model added to the integration is a decision tree. XGBoost can perform the three major gradient boosting techniques, namely, gradient boosting, regularized boosting, and stochastic boosting.

## Training dataset

This dataset is very sparse, including 189,585 samples, of which only 5430 positive samples (known associations verified by experiments) are identified, and the proportion of positive samples is 2.86%. To better evaluate the model, we use the same method as in reference [44] to select negative samples, so we obtain a total of 5430 positive samples and 5418 negative samples, which form a relatively balanced dataset. By combining the feature extraction methods mentioned earlier and merging the structural features, the training dataset is finally formed, which contains 216 features of 10,849 samples.

## Data Availability

The source code and datasets analysed during the current study are available at https://github.com/Changlong2020/ELMDA. All data used in the paper, including the data of miRNA-disease associations, miRNA-target and disease-target interactions, were obtained from current public databases and were cited in the text. The experimentally verified human miRNA–disease associations were downloaded from HMDD database (http://www.cuilab.cn/hmdd), where HMDD V2.0 was used for model training and HMDD V3.2 was used for case studies. The disease semantic similarity was downloaded form http://www.cuilab.cn/files/images/cuilab/misim.zip. The disease-mRNA interactions were obtained from DisGeNET V7.0 (https://www.disgenet.org/downloads) and used to calculate disease functional similarity. The miRNA‒mRNA interaction data downloaded from the miRTarBase database, Release 9.0 (https://mirtarbase.cuhk.edu.cn/~miRTarBase/miRTarBase_2022/php/download.php).
